# A Comprehensive Study on the Degradation Behavior and Mechanism of Expanded Thermoplastic Polyurethane

**DOI:** 10.3390/polym17081033

**Published:** 2025-04-11

**Authors:** Wei Zhao, Shiying Luo, Qing Zhuo, Yuguang Liang, Yuanyuan Li, Hangyu Dong, Liu Qin, Yingru Li

**Affiliations:** 1 Key Laboratory of Green Manufacturing of Super-Light Elastomer Materials of State Ethnic Affairs Commission, Hubei Minzu University, Enshi 445000, China; 202230510@hbmzu.edu.cn (W.Z.);; 2 College of Intelligent Systems Science and Engineering, Hubei Minzu University, Enshi 445000, China; 3 Ningbo GMF New Material Technology Co., Ltd., Ningbo 315000, China

**Keywords:** expanded thermoplastic polyurethane, thermal aging, xenon lamp aging, weathering aging, performance degradation

## Abstract

Expanded thermoplastic polyurethane (ETPU) is used in a wide range of applications due to its excellent properties, but inevitably, aging deteriorates the material properties and shortens service lifetime. This study conducted aging experiments on ETPU to summarize the deterioration trend and provide reliable data. The ETPU underwent three distinct aging protocols: thermal aging for 28 days in a controlled 80 °C environment; xenon lamp aging under continuous UV irradiation (via xenon lamp) at 80 °C for 28 days; and weathering aging through 671 days of outdoor exposure to real-world weather conditions. After various structural characterization and performance tests on the aged ETPUs, the results showed that thermal aging is not the key factor causing the aging of ETPU; the internal structure of ETPU is damaged and the performance rapidly deteriorates under the combined effect of light, heat, and humidity. The special heterogeneous structure gives the sample different internal aging characteristics, and the bead interface becomes a defective site after aging, affecting the overall mechanical properties of the material. In the natural state, the lifetime of ETPU is about two years. Our work will provide valuable data for the study of the aging properties of ETPU and contribute to the prediction of the lifetime of the material.

## 1. Introduction

**Figure 1:**
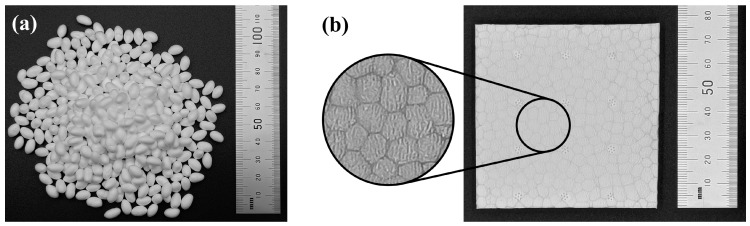
(**a**) Digital photo of ETPU beads; (**b**) ETPU product with special structure.

Expanded thermoplastic polyurethane (ETPU) is a novel foaming material, which is prepared by applying a supercritical carbon dioxide foaming (SCF) process on thermoplastic polyurethane (TPU), and this physical foaming method is green and eco-friendly without hazardous gas emission [1,2,3]. ETPU possesses a variety of advantages, such as low density, good resilience, high abrasion resistance, etc. [4,5]. After the SCF process, ETPU appears in the form of ellipsoid-like beads with the size of several millimeters (Figure 1a), and ETPU beads can be molded into a variety of products with complex structure through the vapor hot press molding process, and they are widely used in the footwear industry, plastic runways, protective gear, and other fields [6,7,8]. The SFC process ensures that the internal pores of ETPU are uniformly and randomly distributed, which gives it isotropic performance [9,10]. Considering that most of the ETPU products are used in outdoor or harsh environments, they are certain to be subjected to humidity, temperature, ultraviolet radiation, and other factors [11,12]. These factors can cause ETPU to age, leading to a variety of degradations, such as oxidation, chain breaking, crosslinking, etc., resulting in structural changes, so that the physical, chemical, and mechanical properties of the material would inevitably deteriorate [13,14], affecting the service life of the material and causing great economic losses [15,16]. Moreover, the structure of ETPU products shows interesting differences compared with other PU foaming material: it is not a uniform foaming material, and boundaries inherited from the ETPU beads can be observed widely (Figure 1b). During the vapor hot press molding process, the surface of the ETPU beads is molten while the inner parts remain in a high-elastic state to keep the beads’ shape. In other words, the boundary regions of ETPU products have experienced the treatment of high-temperature water vapor, while the other regions have not. Different processing histories might bring different aging properties. Therefore, given the wide applications and the unique heterogeneous structure, it is of great importance to investigate the aging of ETPU, to explore the methods used to predict the products’ lifetime, or to help improve the processing techniques for prolonging lifetime.

To fully evaluate the aging properties of ETPU, natural aging and accelerated aging are both essential. Natural aging could reflect the real aging process of polymers, but the very slow reaction rate of natural aging leads to significant limitations in performance testing, which requires the use of an accelerated aging experiment with different conditions to obtain enough data in a much shorter time to predict lifetime [17,18,19]. With the increasing demand for material aging performance evaluation, accelerated aging experiments have been widely adopted by industry and academia [20]. Assis [21] et al. investigated the bending properties of bio-based polyurethane composites after long-term aging in a high-humidity environment. Increased exposure time during aging impaired the interfacial bonding of the composites, thus reducing the bending modulus and bending strength of the materials. And they found that it takes twice as long to age the composites in 100% relative humidity compared to complete immersion in water. Shuai [22] et al. conducted long-term aging experiments on rigid polyurethane foam and proposed a novel random vibration test method to simulate the dynamic stress loads on the material during transportation, and they predicted the service life of the material by its intrinsic characteristic resonance frequency. The results are the same as those of the traditional method by orders of magnitude, and this dynamic random vibration test is more reasonable and convenient. Rybarek [23] et al. placed rigid polyurethanes in hot air and seawater for up to 10 weeks of aging. The results showed that aging deteriorates the appearance, morphology, and mechanical properties of polyurethanes, where the results of foam morphology analysis showed that the aging treatment causes cracking at the edges of the cells, which generates debris that collects at the bottom of the cells, and that this process continues with aging time, resulting in a decrease in the homogeneity of the cell morphology.

In addition, this study summarizes the reported research on aging properties of supercritical foams. Zhang [24] et al. prepared materials with different molecular weights by the microporous foaming of Poly (butylene adipate-co-butylene terephthalate) (PBAT) using supercritical CO_2_. PBAT was placed in organic soil for biodegradation experiments, and the results showed that PBAT had good crystallinity and high bubble density when the content of BT chain segments was 52%, at which time the material showed good degradation properties and dimensional stability. Wei [25] et al. foamed Poly (butylene succinate-butylene terephthalate) (PBST) using supercritical CO_2_ and studied its degradation behavior. They placed the material in sodium hydroxide solution at 60 °C for long-term aging experiments. The results showed that the degradation properties of the foams showed an opposite trend to the mechanical properties with the increase in BT content, and the PBST foams had good degradation and compression properties when the BT content was 60%. Li [26] et al. blended poly (butylene succinate) (PBS) with PBAT and carried out supercritical CO_2_ foaming; they successfully prepared foams with foam density of less than 0.1 g/cm^3^. And the composite foam was subjected to long-term aging experiments; the results show that PBS improved the crystallinity of the composite material but accelerated the degradation rate; in addition, the blended structure ensured the growth of cells, so that the material had better thermal insulation performance. However, there are some limitations in the existing reports. While researchers have studied the aging mechanism of supercritical foams and its degradation of properties in great depth, there are fewer studies on the aging properties of ETPU, a special foaming material. The lack of aging data (e.g., mechanical data, etc.) for ETPU can seriously affect the judgment of material service life as well as limit the application range of ETPU.

In order to solve the above problems, we use the same ETPU samples for both accelerated and weathering aging experiments. The samples are placed in thermal, ultraviolet, and outdoor environments for a long period of aging; then, the samples are tested for changes in appearance, structure, and performance to verify the consistency of the accelerated aging and weathering aging results. Therefore, this work provides reliable data on the changes in the properties of ETPU materials after aging and helps to judge the service life of the materials.

## 2. Experimental Details

### 2.1. Materials

ETPU beads synthesized from polytetrahydrofuran glycol (PTMG) and 4,4′-diphenylmethane diisocyanate (MDI) were purchased from Ningbo GMF New Material Technology Co., Ltd. (Ningbo, China). The performance parameters of ETPU as shown in Table 1.

### 2.2. Aging Process

#### 2.2.1. Sample Preparation

**Figure 2:**
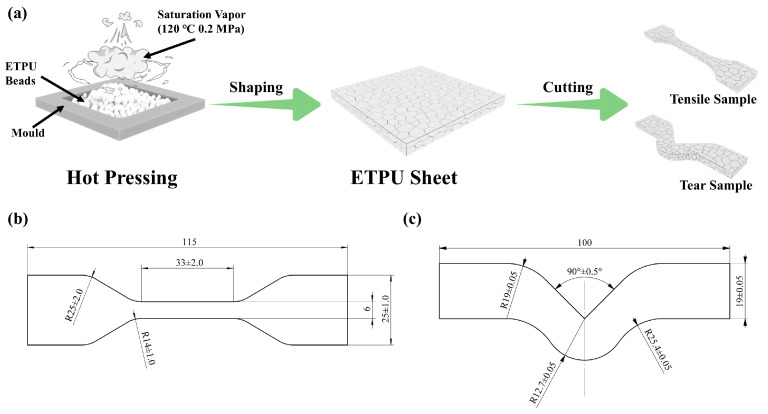
Preparation process and sample dimensions. (**a**) Preparation process of ETPU, (**b**) dimensions of tensile samples, (**c**) dimensions of tear samples.

**Figure 3:**
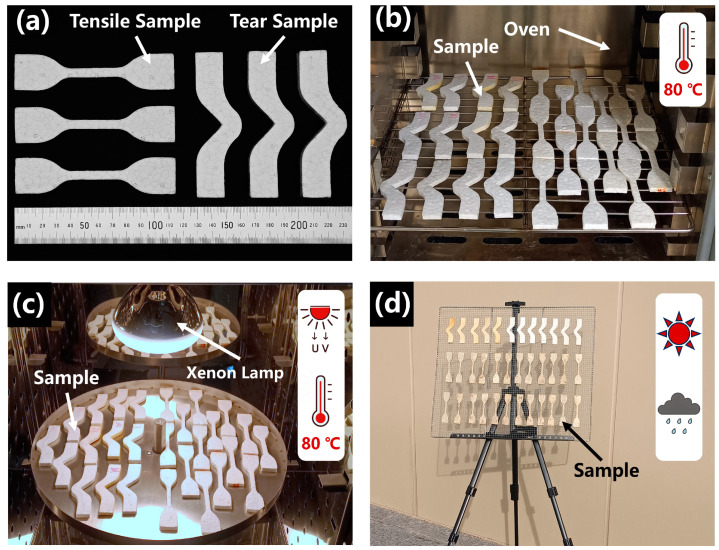
ETPU samples and aging process. (**a**) ETPU tensile samples and tear samples; (**b**) artificial thermal aging experiments; symbol ‘
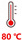
’ in the figure indicates that the samples were exposed to 80 °C for 7, 14, 21, and 28 days; (**c**) artificial xenon lamp aging experiments; symbols ‘
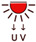
’ and ‘
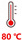
’ in the figure indicate that the samples were exposed to 80 °C for 7, 14, 21, and 28 days and illuminated by UV light at the same time; (**d**) weathering aging experiments; symbols ‘
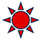
’ and ‘
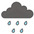
’ in the figure indicate that the samples have been in a natural environment for 276, 377, and 671 days and have been subjected to the most realistic weather changes.

ETPU beads were made into sheets with a thickness of 5 mm by using the vapor hot press molding process, with the temperature of the vapor set at 120 °C and a pressure of 0.2 MPa. The complete process of sample preparation is shown in Figure 2a. According to GB/T 528-2009, the ETPU sheet was cut into several dumbbell-shaped tensile samples to test the tensile properties of the material, as shown in Figure 2b. According to GB/T 529-2008, the ETPU sheet was cut into several right-angle shaped samples to test the tear strength of the material, as shown in Figure 2c. In order to minimize errors, at least five identical samples were taken for each tensile and tear test. The vapor compression molding process allows the ETPU beads to be extruded and adhered to each other to form a special texture; the ETPU sample is shown in Figure 3a.

#### 2.2.2. Artificial Thermal Aging Process

High temperature is one of the major factors leading to the aging of macromolecules. Thermal aging can accelerate the movement of chain segments within the polymer, and active free radicals will be generated in the process, which will make the chain segments break and ultimately make the macromolecule degraded or crosslinked. The ETPU samples were placed in a constant-temperature blast drying oven (DHG-9035A, Shanghai Bluepard instruments Co., Ltd., Shanghai, China), and the temperature was set to 80 °C. The samples were subjected to artificial thermal aging experiments for 7, 14, 21, and 28 days, respectively (Figure 3b).

#### 2.2.3. Artificial Xenon Lamp Aging Process

Ultraviolet light (UV) and near-infrared light (NIR) causes chain breakage of polymer compounds, which is one of the main factors leading to degradation of polymers by aging. Xenon lamp aging has been proven to be reliable as an accelerated aging experiment. The ETPU samples were placed in an xenon lamp aging test machine (LY-605A, Dongguan LIYI Experiment Instrument Co., Ltd., Dongguan, China), in which the radiation sources, such as UV and visible and NIR light, were supplied by a xenon lamp (OSRAM, Munich, Germany) with a power of 300 W. Samples were located 20 cm away from the light source; the average irradiation intensity per hour in the UV wavelength range of 315–400 nm was 13.6 W/m^2^, the average irradiation intensity per hour in the visible range of 380–780 nm was 41.4 W/m^2^, and the temperature in the aging machine was set at 80 °C (Figure 3c). The samples were subjected to xenon lamp aging tests for 7, 14, 21, and 28 days under the same experimental environment.

#### 2.2.4. Weathering Aging Process

Exposing the samples to atmospheric conditions allows the samples to age in real environments; the most accurate aging information is obtained from this experimental method. The ETPU samples were placed in a clearing away from the shade of trees and buildings and were subjected to the most realistic weather conditions (Figure 3d). The experimental site was located in Enshi, China (109.5103 E, 30.3004 N, and 430 m above sea level); the height of the samples from the ground was 1 m, and the angle of inclination formed between the horizontal plane of the samples and the ground was 45°; they were facing due south, so that the samples were exposed to the maximum amount of solar UV radiation. The experiment started on 17 July 2022 and ended on 24 July 2024; the specific state of the natural environment during the experiment is shown in Table 2.

### 2.3. Characterization

Scanning electron microscopy (SEM) was used to observe the microscopic morphology of the fracture surface of the ETPU, using a secondary electron detector (SE) and obtaining morphological images at an accelerating voltage of 5 keV (MIRA LMS, TESCAN, Brno, Czechia). Attenuated total refraction–Fourier transform infrared spectroscopy (ATR-FTIR) was used to demonstrate the internal chemical bonding changes of ETPU during the aging process (Nicolet iS20, Thermo Fisher Scientific, Waltham, MA, USA). An electronic universal testing machine was used to test the tensile properties of ETPU (TY8000A, Tian Yuan Test Instrument, Yangzhou, China), the tensile rate was set to 50 mm/min, and the strain growth rate during tensile testing was 0.0253 s^−1^. The stereomicroscope was used to observe the state of the appearance of the samples after aging (T2-HD206, Shenzhen AOSVI Optical Instrument Co., Ltd., Shenzhen, China). Thermogravimetric analysis (TGA) was used to test the changes in thermal behavior of the ETPU materials during aging (EXSTAR TG/DTA6300, SII, Chuo City, Japan). The samples were placed in an alumina crucible and heated from 30 °C to 500 °C with the heating rate set at 10 °C/min, and the flow rate of nitrogen was set at 100 mL/min.

### 2.4. Color Measurements

The color change of the ETPU surface was tested using a portable colorimeter with a light source type of D65 and a light source angle of 10 degrees, and the color change was quickly evaluated using the CIELAB color system (CR30, Hangzhou Color Spectrum Technology Co., Ltd., Hangzhou, China). There are three parameters (L*, a*, b*) used by this color system to represent all colors, where L* represents luminance, a* represents red to green, and b* represents blue to yellow. The overall color change of the sample would be represented using the parameter ∆E*_ab_, calculated as ∆E*_ab_ = [(L*_1_ − L*_2_)^2^ + (a*_1_ − a*_2_)^2^ + (b*_1_ − b*_2_)^2^]^1/2^, where L*_1_, a*_1_, and b*_1_ denote the color parameters of the unaged sample, and L*_2_, a*_2_, and b*_2_ denote the color parameters of the aged sample. Seven consecutive readings were taken randomly on the surface of the specimen, and the mean values of L*, a*, b* were calculated.

## 3. Results and Discussion

### 3.1. Colorimetric Characterization of ETPU Surface

**Figure 4:**
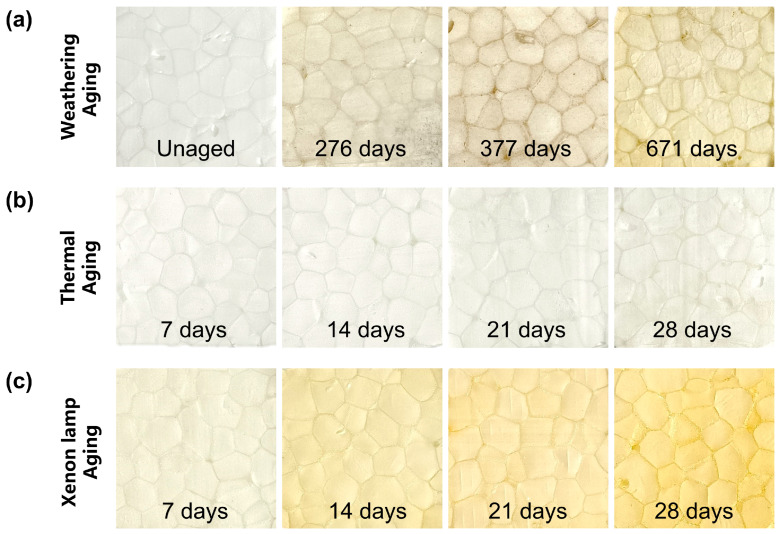
Color comparison of ETPU samples after different aging treatments. (**a**) Weathering aging process; (**b**) artificial thermal aging process; (**c**) artificial xenon lamp aging process.

ETPU would turn yellow under high temperature and UV irradiation for a long period of time, and the color of ETPU would gradually deepen with the aging time, which is the most intuitive judgment of the aging of the material and the degree of aging that has occurred, reflecting the chemical structure changes due to auto-oxidization, cyclization, carbonylation, etc. Moreover, the color changes of polymer material often show a strong correlation with the degradation of mechanical properties. The color comparison of ETPU samples under three aging environments are shown in Figure 4. The unaged ETPU surface appears white and shiny, while the yellowing gradually deepens and the surface gloss degrades with the increase in weathering aging time. After 671 days of aging, the surface of the material showed obvious wrinkles and cracks (Figure 4a). With ETPU under the thermal aging environment, the surface color change is not obvious; after aging 28 days, there is only a slight yellowing of the surface, and no obvious cracks appear (Figure 4b). In contrast, the surface of the samples showed very obvious changes after xenon lamp aging. After 14 days of the xenon lamp aging process, the degree of surface yellowing was similar to that after 671 days of weathering aging. After 28 days, the surface of the ETPU samples showed an amber color (Figure 4c).

The UV light with a wavelength of 330–340 nm induced a photo-Fries rearrangement of the aromatic groups, resulting in the production of chromophores and an auxochrome group [27,28,29]. The total amount of radiation of the weathering aging and xenon lamp aging was calculated, and the cause of yellowing in the ETPU aging was analyzed. The annual average total solar radiation in Enshi city [30] was about 4500 MJ/m^2^, of which the UV irradiance was 9.9 W/m^2^, and the UV irradiance of the xenon lamp was 13.6 W/m^2^. The weathering aging and xenon lamp aging processes both provide sufficient UV light for the aging of ETPU, which makes the yellowing of the samples very obvious, but xenon lamp aging can provide a large amount of UV radiation in a short period of time, and the sustained high temperature of 80 °C will also promote the further aging of the samples, which makes the yellowing of the ETPU samples more obvious. In contrast, the yellowing of the ETPU samples is not obvious due to the absence of UV light during thermal aging. In addition, during the weathering aging process, water molecules in the environment will penetrate into the ETPU and cause swelling, and they interact with the hydrogen bonds within the material, affecting the stability of the hydrogen bonds and causing them to break [31,32]. The changing temperature in the environment will also lead to the degradation of ETPU. All of these factors can cause cracks to form on the surface of the material. After weathering aging for 671 days, the images show more pronounced cracks and wrinkles.

**Figure 5:**
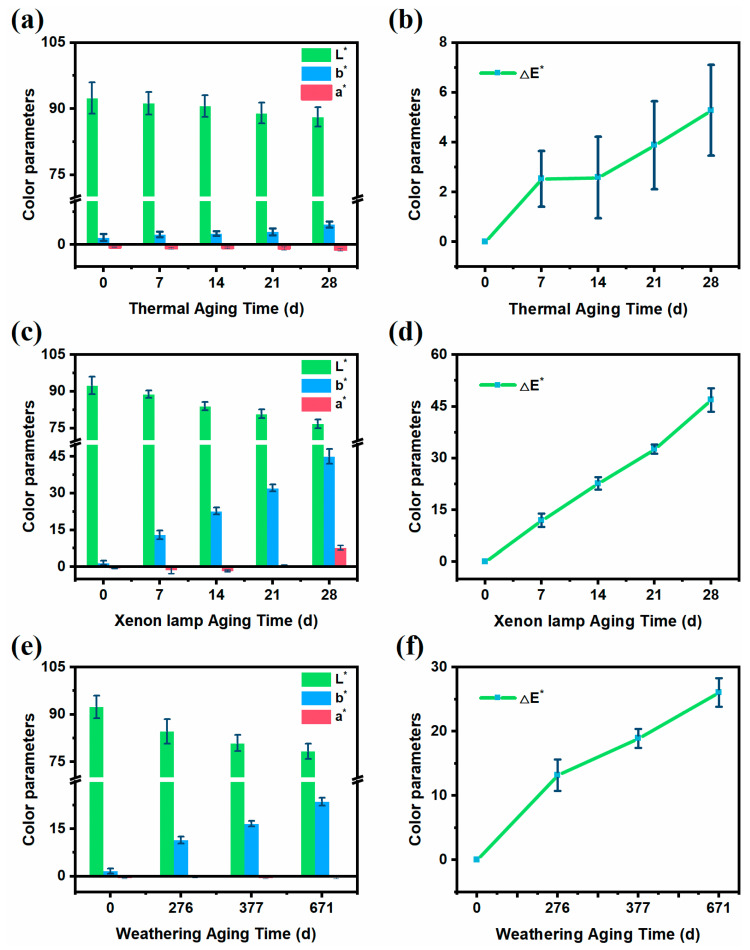
CIE lab parameters of ETPU. Artificial thermal aging process for (**a**) color parameter variation and (**b**) overall color variation; artificial xenon lamp aging process for (**c**) color parameter variation and (**d**) overall color variation; weathering aging process for (**e**) color parameter variation and (**f**) overall color variation.

The CIELAB color parameters were used to specifically quantify the degree of yellowing of ETPU. The unaged sample was used as the reference standard, the values of L*, a*, and b* on the surface of the aged sample were measured, respectively, and the difference ∆E*_ab_ from the reference standard was calculated; the results are shown in Figure 5. During the thermal aging process, the overall changes in L* and b* on the ETPU surface were not significant, and the color difference (∆E*_ab_) between the ETPU surface and the unaged sample was slight (Figure 5a,b), implying that the surficial aging caused by a single thermal factor is not severe. In the process of xenon lamp aging, with the prolongation of UV illumination, the b* value on the surface of ETPU showed a positive increasing trend, and after 28 days of xenon lamp aging, the b* value was about 30 times that of the unaged samples; in addition, the luminance value (L*) decreased to a certain extent, and the change in the ∆E*_ab_ value was very obvious (Figure 5c,d), nearly nine times that in thermal aging experiments, showing that the strong irradiation of xenon arc light could significantly accelerate the aging rate. In the weathering aging process, the trend of color parameter changes was similar to that of xenon lamp aging (Figure 5e,f). At 671 days, the ∆E*_ab_ was similar to that of the sample in xenon lamp aging on the 14th day, agreeing with the color change in Figure 4, but with more wrinkles on the surface, suggesting that humidity promotes the hydrolysis of ETPU.

Positive changes in color parameters were observed for all samples, indicating that yellowing occurred in all samples. The insignificant change in color parameters during thermal aging is due to the relatively low temperature of 80 °C, which allows a lower rate of thermal decomposition and the production of fewer chromophores [33]. The total amount of UV radiation produced during weathering aging for 671 days is about 580 MJ/m^2^, while the total amount of UV radiation produced after 28 days of xenon lamp aging is only 35 MJ/m^2^. Although the total amount of UV radiation produced by xenon lamp aging is much lower than weathering aging, due to the effect of thermal aging, the increase in the rate of free radical auto-oxidation promotes the production of chromophores, making the yellowing more obvious.

Generally speaking, the thermal factor alone did not cause significant deterioration of the ETPU samples, but the addition of UV irradiation accelerated the deterioration of the sample surfaces considerably. In addition to this, humidity also triggers hydrolysis reactions, making the surface deterioration of the samples more pronounced.

### 3.2. Chemical Structure Variation During Aging of ETPU

**Figure 6:**
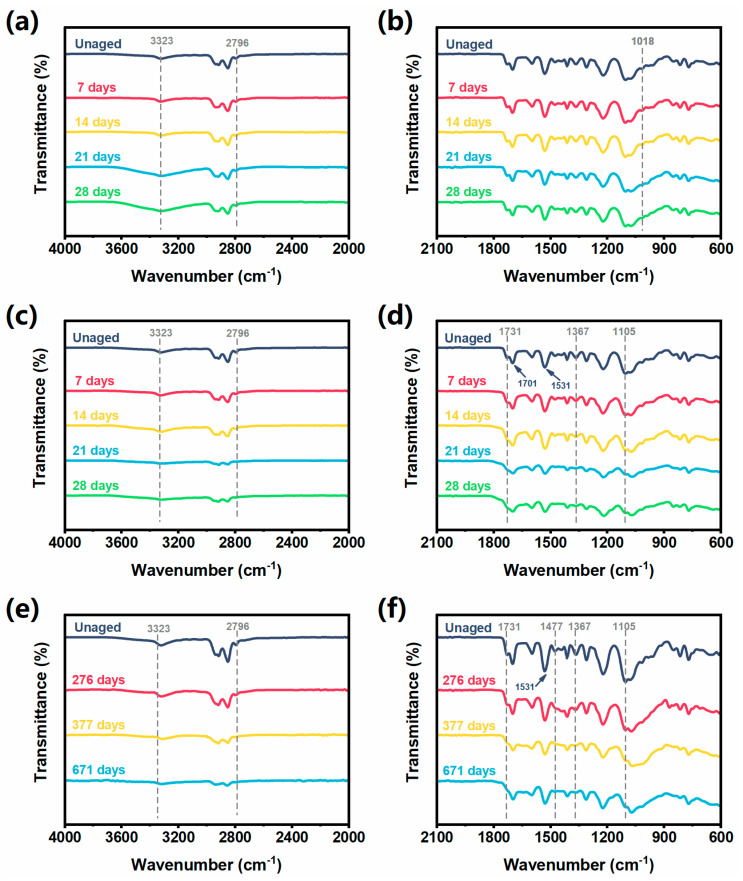
FTIR spectra of ETPU samples in (**a**) 4000–2000 cm^−1^ band, (**b**) 2100–600 cm^−1^ band after thermal aging for 7, 14, 21, and 28 days; FTIR spectra of ETPU samples in (**c**) 4000–2000 cm^−1^ band, (**d**) 2100–600 cm^−1^ band after xenon lamp aging for 7, 14, 21, and 28 days; FTIR spectra of ETPU samples in (**e**) 4000–2000 cm^−1^ band, (**f**) 2100–600 cm^−1^ band after weathering aging for 276, 377, and 671 days.

The ETPU samples after aging treatment were analyzed by FTIR, and the results are shown in Figure 6. In order to exclude the influence of environmental factors, all the samples after aging were stored and sealed before FTIR testing. In the infrared spectrum, polyurethane has several characteristic peaks [34,35,36]. A broad peak at 3323 cm^−1^ is generated by the stretching vibration of the hydrogen bond in the intermolecular state. The multiple peaks at 2700–2950 cm^−1^ are generated by the stretching vibration of C-H bonds. The two peaks located at 1531 cm^−1^ and 1701 cm^−1^ are characteristic peaks arising from the vibration of the secondary amide structure in the carbamate group, where 1531 cm^−1^ is generated by the N-H bond bending vibration, and 1701 cm^−1^ is generated by the C = O double bond stretching vibration. The characteristic peak at 1413.47 cm^−1^ is generated by the C-N bond stretching vibration. The multiple peaks in the 900–1350 cm^−1^ fingerprint region are generated by C-O bond stretching vibration. The characteristic peaks at 1598 cm^−1^ are generated by the C = C vibration in the aromatic ring and at 814 cm^−1^ by the out-of-plane bending vibration of the C-H bond of the 1,4-disubstituted aromatic ring, and the two sets of characteristic peaks undergo absorbance coupling.

There was no major structural change observed in the thermal aging of the ETPU samples, and there were mainly two characteristic peaks in the infrared spectra that changed significantly (Figure 6a,b). With the increase in thermal aging time, the band of the characteristic peak of the N-H bond at 3323 cm^−1^ gradually widens, which is due to the deterioration of the polymer by thermal aging, the destruction of hydrogen bonding, and the appearance of other types of interactions, making the absorption peak here broader. The absorption peak at 1018 cm^−1^ shows a slight decrease in intensity due to the loss of carbonyl group (C-O) by thermal aging [37].

Figure 6c,d shows the FTIR spectra of the ETPU after UV light radiation. It is noteworthy that no new peaks appeared in the spectra after UV irradiation, but there were old peaks that disappeared, which means that UV irradiation destroyed some groups in the ETPU. The two peaks located at 1530.94 cm^−1^ and 1700.92 cm^−1^, as well as the intensity of the characteristic peaks in the region of 900–1350 cm^−1^, showed a decreasing trend, which was attributed to the fact that the UV light induced the generation of peroxyl radicals inside the ETPU, which destroyed the carbamate bonds in the molecular chain segments. In addition, the destruction of the carbamate bond will lead to the destruction of part of the intermolecular hydrogen bonding as well, resulting in a decrease in the peak area of the broad peak located at 3323.20 cm^−1^. However, there is an increase in the width of the peak at 3323.20 cm^−1^, which is due to new interactions within the molecule as a result of the breakage of the C-N bond in the carbamate group [38]. The intensity of the multiple peaks in the region of 2700–2950 cm^−1^ decreased significantly, and the characteristic peak at 2796 cm^−1^ basically disappeared after 28 days of xenon lamp aging, while the characteristic peak at 1105 cm^−1^ also decreased gradually. The peaks in the 2700–2950 cm^−1^ region and at 1105 cm^−1^ belong to the telescopic vibration of the C-H bond and the vibration peak of C-O in the polymer chain segment, respectively; so, it can be reasonably deduced that UV light irradiation destroys the methyl, methylene, and carbonyl structures in the structure of the chain segment of the ETPU, which results in the breakage of the chain segment [11].

During the weathering aging process, ETPU is subjected to a variety of factors (e.g., water, UV light, and changing temperatures), which induces it to undergo aging. The intensity of all the characteristic peaks in the infrared spectrum of the ETPU decreased or even disappeared, indicating that degradation must have occurred inside the sample. The characteristic peaks located at 1105 cm^−1^ show a decreasing trend in intensity, which is caused by the C-O breakage within the carbamate bond (Figure 6e,f). The decreasing trend of the characteristic peaks in the region of 2700–2950 cm^−1^ is very obvious, and the signal of the characteristic peaks in this region becomes very weak after 671 days of weathering aging, indicating that a large number of C-H bonds have been broken. The characteristic peaks located at 1731 cm^−1^, 1531 cm^−1^, and 1477 cm^−1^ are generated by the secondary amide structure in polyurethane, and the peak intensities are significantly reduced, indicating that a large number of polyurethane structures are destroyed. The characteristic peaks at 1367 cm^−1^ and 2796 cm^−1^ are generated by the methylene bending vibration and methyl stretching vibration, respectively, and the near disappearance of these two characteristic peaks indicates that the internal chain segment of the polymer is broken. The intensity of the peak at 3323 cm^−1^ generated by the stretching vibration of intermolecular hydrogen bonding also decreases gradually, which also proves the destruction of the polyurethane molecular structure [13].

### 3.3. Evaluation of Thermal Properties of ETPU

**Figure 7:**
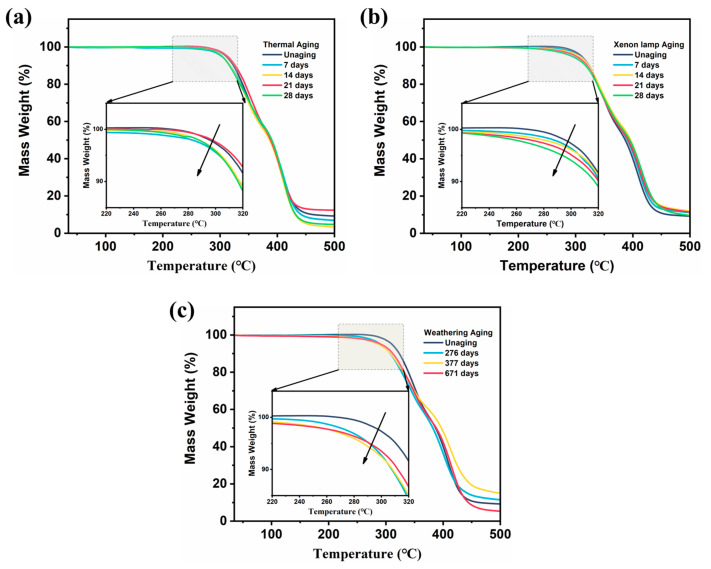
ETPU thermal performance evaluation. (**a**) TG curve of ETPU after artificial thermal aging process; (**b**) TG curve of ETPU after artificial xenon lamp aging process; (**c**) TG curve of ETPU after weathering aging process.

The thermal stability of the samples before and after aging was investigated, and the TG curves of the samples are shown in Figure 7. The TG curves of all the samples show similar trends, and the curves can be divided into three stages. The first stage (100–250 °C) is mainly attributed to the removal of adsorbed water and bound water from the material, and the weight loss in this stage is very slight (about 1–2%). The weight loss in the second stage (250–350 °C) is mainly due to the decomposition of the urethane groups and polyether chain segments in the ETPU. The decomposition products in this stage include carbon dioxide, amines, and other small molecules, and the overall weight loss reaches about 42%, at which point the polyurethane structure has been significantly damaged. In the third stage (350–450 °C), the polyether chain segment undergoes further degradation until the chemical bond is completely broken, at which point more small molecules will be produced; the weight loss rate in this stage is about 48%. With a further increase in temperature, the polymer will gradually carbonize and form a black carbonaceous residue at the bottom of the crucible, at which point only about 10% of the material mass remains [39,40].

Obviously, the aging treatment affects the thermal stability of ETPU. After the thermal aging process, the initial thermal decomposition temperature of the ETPU samples decreased, and the thermal decomposition rate at each stage did not change significantly. This is due to the fact that 80 °C did not cause excessive damage to the internal structure of ETPU; therefore, the thermal properties did not show a significant attenuation (Figure 7a). UV irradiation reduced the initial thermal decomposition temperature of the samples and caused a decrease in the rate of thermal decomposition in the second stage. UV light caused a large number of chemical bonds within the ETPU to break, producing many oligomers and small molecules that are easily degraded, leading to a decrease in thermal stability (Figure 7b). During the weathering aging process, the initial decomposition temperature of ETPU in the first stage significantly decreases. Thermal stability is reduced by the breakdown of internal chemical bonds; in addition, the sample absorbs water from the environment and undergoes swelling, which alters the altered cell structure, thereby affecting the thermal conductivity and thermal decomposition behavior of the material (Figure 7c).

### 3.4. Degradation of Mechanical Properties of ETPU During Aging Process

In this section, we investigate the changes in the mechanical properties of ETPU when exposed to different aging environments over a long period of time. Tear strength (T), which is a measure of the ability of a polyurethane foam to resist a tearing load, is calculated using T = F/D, where F denotes the tensile load at which the sample breaks, and D denotes the thickness of the sample; each sample is tested five times [41].

The tear strength values of the ETPU after the aging process are shown in Table 3. The tear strengths of the ETPUs were all decreased to different degrees after aging treatment. The decreasing trend of the tear strength of the ETPU during thermal aging is not obvious, and after 28 days, it decreases from 12.23 N/mm to 11.51 N/mm. However, the tear strength after xenon lamp aging shows the trend of increasing and then decreasing; eventually, it decreases to 9.37 N/mm. Long-term weathering aging will destroy the internal structure of the material; the tear strength decreases to 8.46 N/mm after 671 days, and the decreasing rate reaches 30%.

Prolonged aging causes chemical bonds within ETPU to break and intermolecular forces to decrease, making the microstructure weaker. When the tear force is applied, the material is more likely to produce cracks, which reduces the tear strength. In addition, in the early stage of xenon lamp aging, crosslinking reaction occurs within the ETPU, which will improve the overall structure of the material, making the tear strength increase, but in general, the tear strength of the material shows a tendency to decrease.

Tensile strength is another important criterion of the mechanical properties of ETPU; so, the trend needs to be discussed separately, and the tensile strength values are shown in Table 4. The tensile strength of the ETPU is 1.5 MPa (unaged sample), and with the increase in aging time, the tensile strength shows an overall decreasing trend and decreases to 0.61 MPa after 671 days of weathering aging, with a decay rate of 59.3%. After the thermal aging process, the tensile strength decreased to 1.36 MPa after 28 days, and the degree of decrease was not obvious. In addition, the tensile strength after 28 days of xenon lamp aging process was similar to that after 377 days of weathering aging in the dry environment, which proved that the mechanical properties of the ETPUs decreased rapidly after continuous exposure to high-intensity UV irradiation.

**Figure 8:**
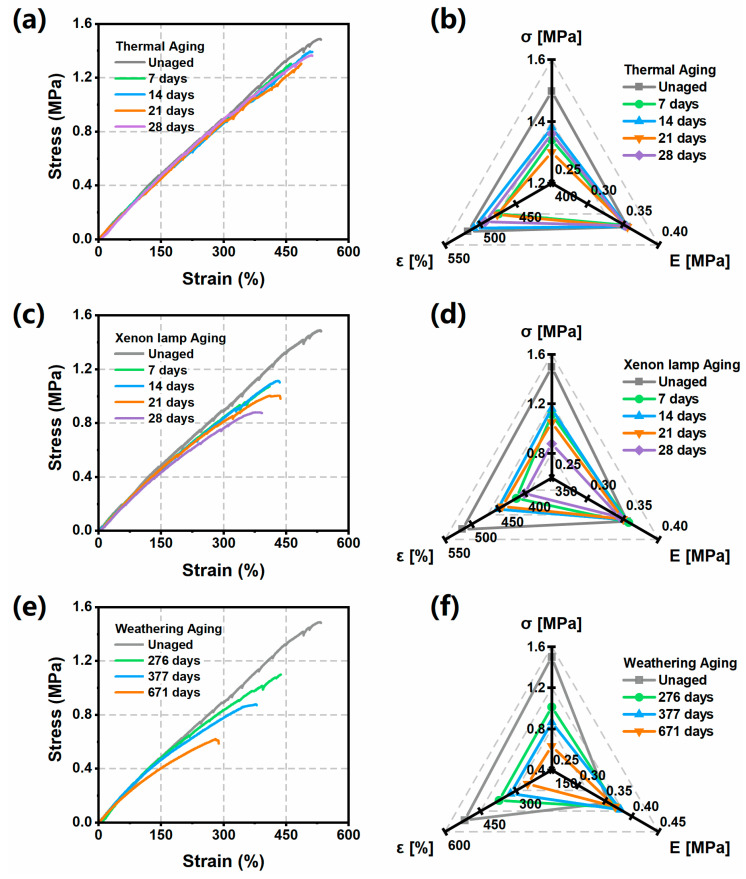
Mechanical properties of ETPU samples. (**a**) Stress–strain curve and (**b**) tensile property parameters of ETPU after artificial thermal aging process; (**c**) stress–strain curves and (**d**) tensile performance parameters of ETPU after artificial xenon lamp aging process; (**e**) stress–strain curves and (**f**) tensile performance parameters of ETPU after weathering aging process. In the figure, σ denotes tensile strength, E denotes tensile modulus, and ε denotes elongation at the break.

The trends of tensile strength, tensile modulus, and elongation at the break were comprehensively evaluated, as shown in Figure 8. The thermal aging process did not have an excessive effect on the tensile properties of the ETPU, and the decreasing trend of tensile strength, tensile modulus, and elongation at the break was not obvious (Figure 8a,b). This is attributed to the relatively stable chemical chain structure of the polyurethane, and 80 °C is not enough to cause significant changes in the chemical bonds and degradation of the material. Moreover, a crosslinking reaction occurs in the material at the early stage of aging, and with the continuation of time, the crosslinking reaction will be balanced with the chain segment breakage, which ultimately keeps the mechanical properties of the material in a relatively stable state. In contrast, after UV irradiation, the mechanical properties of the ETPU showed a very obvious decline (Figure 8c,d). This is due to the fact that UV irradiation leads to the breakage of C-H and C-C bonds in polyurethane materials and the formation of free radicals, which in turn triggers a series of polymer chain breakage reactions. UV irradiation is also accompanied by oxidative reactions, which can lead to a decrease in material properties. The decrease in ETPU performance after weathering aging is even more obvious. After 671 days of aging, the tensile strength is only about 40% of the unaged sample, but the tensile modulus is slightly increased (Figure 8e,f). The reason is that the crosslinking reaction in the early stage of aging will make the surface of ETPU harden, increasing its stiffness and tensile modulus. However, the changing environment makes the material surface crack, which becomes a defective part of the material, so that the material is more prone to stress concentration, thus reducing the tensile strength and elongation at the break of the material.

GB/T 20028 is used to derive the lifetime of thermoplastic elastomer materials. The numerical change in the mechanical properties of the sample (e.g., tensile strength, elongation at the break, etc.) is considered as a function of time. The time taken for the mechanical properties to decay to 50% of the original properties is the lifetime of the material. The tensile strength of the unaged ETPU was 1.5 ± 0.1 MPa, and the elongation at the break was 515 ± 25%; so, the threshold values of the mechanical properties were 0.75 MPa and 258%, respectively. After weathering aging for 671 days, the tensile strength of the ETPU drops to 0.61 MPa, which is already completely below the threshold, and the elongation at the break drops to 287%, near the performance threshold. At this time, the ETPU can be considered to have reached the end of its lifetime. Therefore, in natural conditions, the lifetime of ETPU is about 2 years.

### 3.5. Fracture Morphology Analysis of ETPU Tensile Samples

**Figure 9:**
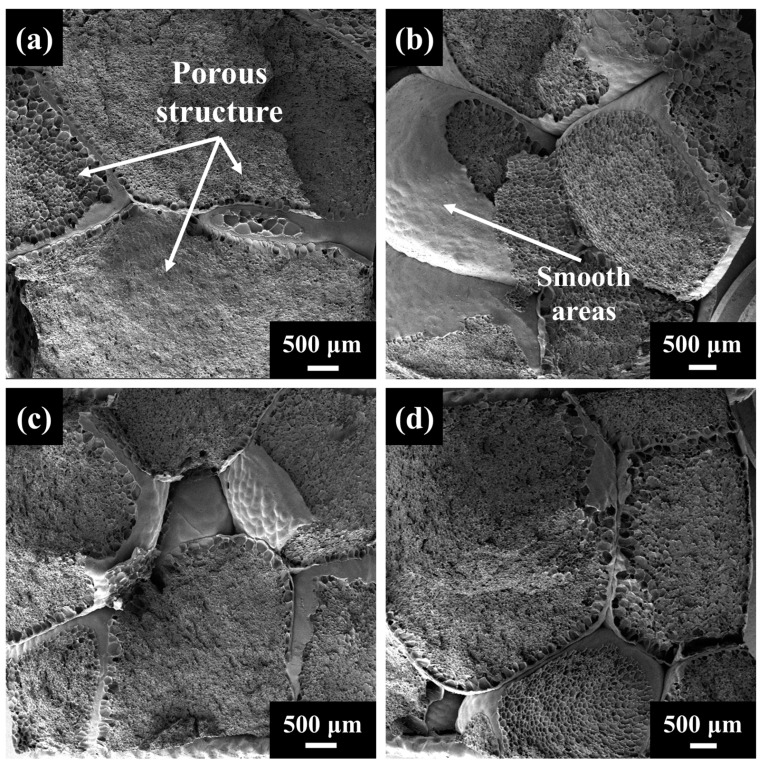
SEM image of ETPU tensile sample at the fracture surface. (**a**) Unaged sample; (**b**) sample after 671 days of weathering aging process; (**c**) sample after 28 days of thermal aging process; (**d**) sample after 28 days of xenon lamp aging process.

During the vapor hot press molding process, the thermal history of the ETPU bead surface is different from that of the interior, which can make the ETPU surface have different mechanical properties from the interior, causing the ETPU bead interface to become an area of aging weakness. The fracture surface morphology of the tensile samples was observed using SEM, as shown in Figure 9.

The SEM images clearly demonstrate that the ETPU tensile samples have different morphologies in their fracture surface after undergoing aging treatments. The fracture surface of the unaged sample shows a large number of uneven areas, and a large number of porous structures can be observed (Figure 9a), which is due to the fact that the ETPU beads tear directly from the middle area when subjected to axial tensile force and show the porous structure inside the beads. The middle region of the beads is the perfect fracture location, where the beads are subjected to the maximum tensile force, and at this time, the mechanical strength of the material is at its optimal state. Therefore, for unaged samples, the boundary area between the beads is not a defective part of the ETPU. With the weathering aging experiments carried out, smooth areas gradually appeared in the ETPU fracture surface (Figure S1). After the weathering aging process for 671 days, a large number of smooth areas appeared in the section (Figure 9b), which indicated that the damaged areas were actually boundary areas between the ETPU beads, which meant that these boundary areas were more aged than the inside of the ETPU beads, thus making the boundary areas mechanically defective. When the sample was subjected to axial tension, it would break from these weak areas. This can cause the mechanical properties of the ETPU to deteriorate substantially.

Compared with the unaged samples, a small number of smooth areas begin to appear in the fracture surface of the ETPU after thermal aging and xenon lamp aging treatments (Figure 9c,d, Figures S2 and S3); this is due to the rapid destruction of the chemical bonds within the material in a short period of time, which gradually begins to appear in weak areas and reduces the mechanical properties of the material. In addition, excessive humidity can also hydrolyze the material and cause deterioration of the properties. During both thermal and xenon lamp aging, the ETPU samples were in a dry environment, while weathering aging had the effect of humidity as well; so, after 671 days of the weathering aging process, more weak areas appeared in the fracture surface.

## 4. Conclusions

The ETPU samples fabricated via vapor hot press molding were subjected to three aging processes. The results are as follows: (1) Xenon lamp aging under dry conditions for 14 days induced surface yellowing comparable to 671-day natural weathering. Despite lower cumulative UV dosage in xenon lamp aging, thermal activation accelerates radical auto-oxidation rates, promoting chromophore formation and intensified discoloration. In addition, weathering-induced humidity triggered ETPU surface hydrolysis, generating macroscopic folds. (2) Mechanical testing revealed that the 28-day xenon lamp-aged specimens exhibited equivalent property degradation to the 377-day weathered samples. This accelerated deterioration arises from UV-induced scission of critical chemical bonds, generating chain segment defects that reduce tensile/tear strength through localized stress concentration. (3) Morphological analysis identified interfacial heterogeneity in the ETPU sheets due to differential processing histories between particle boundaries and internal. Weathering preferentially degraded these interfaces, transforming them into mechanical weak points. In conclusion, synergistic UV–thermal–humidity effects drive rapid performance decay, and ETPU reaches its lifetime in about two years in the natural state. We believe this study will provide valuable data on the aging properties of ETPU and help predict the lifetime of the material, which will broaden the field of use of the material.

## Figures and Tables

**Table 1:** Performance parameters of ETPU.

Characteristics	Unit	Measured Value	Method
Density	g/cm^3^	0.18	GB/T 533-2008
Bead diameter	mm	6	
Shore hardness	C	38	GB/T 531.1-2008
Tensile strength	MPa	1.5	GB/T 1040.1-2018
Tensile modulus	MPa	0.35	GB/T 1040.1-2018
Elongation at break	%	530	GB/T 1040.1-2018
Tear strength	N/mm	12	GB/T 529-2008
Resilience	%	60	GB/T 1681-2009

**Table 2:** Meteorological data during weathering aging.

Date	Avg High (°C)	Max Temp (°C)	Avg Low (°C)	Min Temp (°C)	Total Precipitation (mm)	Medial Humidity
July 2022	35	38	23	19	144.42	78.16%
August 2022	37	41	26	18	51.14	70.35%
September 2022	28	37	19	14	80.56	74.16%
October 2022	23	34	14	9	72.03	73.96%
November 2022	19	26	10	1	77.17	74.51%
December 2022	11	15	3	0	41.81	72.33%
January 2023	8	19	2	−1	49.34	73.03%
February 2023	10	17	5	2	72.87	75.20%
March 2023	18	27	9	4	97.57	78.35%
April 2023	24	33	13	8	203.96	75.33%
May 2023	24	35	17	13	312.49	81.83%
June 2023	27	35	20	18	210.52	79.12%
July 2023	30	37	23	20	258.48	78.16%
August 2023	32	37	22	16	155.46	75.26%
September 2023	29	36	20	16	203.05	75.36%
October 2023	22	28	14	11	176.28	78.91%
November 2023	17	27	10	6	157.07	76.96%
December 2023	11	19	5	0	92.24	72.29%
January 2024	10	19	5	0	125.21	76.74%
February 2024	9	21	4	0	141.61	75.93%
March 2024	17	30	10	3	124.97	74.56%
April 2024	23	33	16	12	221.51	78.13%
May 2024	27	35	18	14	274.54	75.38%
June 2024	28	38	21	17	240.5	77.69%
July 2024	31	38	24	21	398.73	80.83%

**Table 3:** Tear strength of ETPU at different stages after three aging processes.

**Aging Process**	**Tear Strength (N/mm)**	**Aging Time (Days)**				
		**0**	**7**	**14**	**21**	**28**
Thermal aging	$\bar{x}$	12.23	12.06	12.28	11.55	11.51
	s	0.43	0.60	0.48	0.57	0.66
Xenon lamp aging	$\bar{x}$	12.23	12.16	12.71	10.68	9.37
	s	0.43	0.43	0.56	0.46	0.69
**Aging Process**	**Tear Strength (N/mm)**	**Aging Time (days)**				
		**0**	**276**	**377**	**671**	
Weathering aging	$\bar{x}$	12.23	11.71	9.46	8.46	
	s	0.43	0.42	0.47	0.48	

**Table 4:** Tensile strength of ETPU at different stages after three aging processes.

**Aging Process**	**Tensile Strength (MPa)**	**Aging Time (Days)**				
		**0**	**7**	**14**	**21**	**28**
Thermal aging	$\bar{x}$	1.50	1.33	1.38	1.31	1.36
	s	0.07	0.06	0.08	0.05	0.09
Xenon lamp aging	$\bar{x}$	1.50	1.11	1.16	1.05	0.87
	s	0.07	0.05	0.05	0.03	0.04
**Aging Process**	**Tensile Strength (MPa)**	**Aging Time (days)**				
		**0**	**276**	**377**	**671**	
Weathering aging	$\bar{x}$	1.50	1.01	0.86	0.61	
	s	0.07	0.09	0.06	0.07	

## Data Availability

The data presented in this study are available on request from the corresponding author. The data are not publicly available due to [The original contributions presented in the study are included in the article/Supplementary Materials, further inquiries can be directed to the corresponding authors].

## Supplementary Materials

The following supporting information can be downloaded at: https://www.mdpi.com/article/10.3390/polym17081033/s1, Figure S1: SEM image of the fracture surface of the ETPU tensile sample subjected to weathering aging process. (a) unaged sample; (b) aging 276 days; (c) aging 377 days; (d) aging 671 days; Figure S2: SEM image of the fracture surface of the ETPU tensile sample subjected to artificial thermal aging process. (a) aging 7 days; (b) aging 14 days; (c) aging 21 days; (d) aging 28 days; Figure S3: SEM image of the fracture surface of the ETPU tensile sample subjected to artificial xenon lamp aging process. (a) aging 7 days; (b) aging 14 days; (c) aging 21 days; (d) aging 28 days.
