# Supplementary material for: A Comprehensive Study on the Degradation Behavior and Mechanism of Expanded Thermoplastic Polyurethane
Source: Polymers (Basel). 2025 Apr 11;17(8):1033. doi: 10.3390/polym17081033 (PMC12030128; doi:10.3390/polym17081033)
Supplement: Supplementary file 1 [file polymers-17-01033-s001.zip › polymers-3545851-supplementary.pdf]

**Supplementary File**

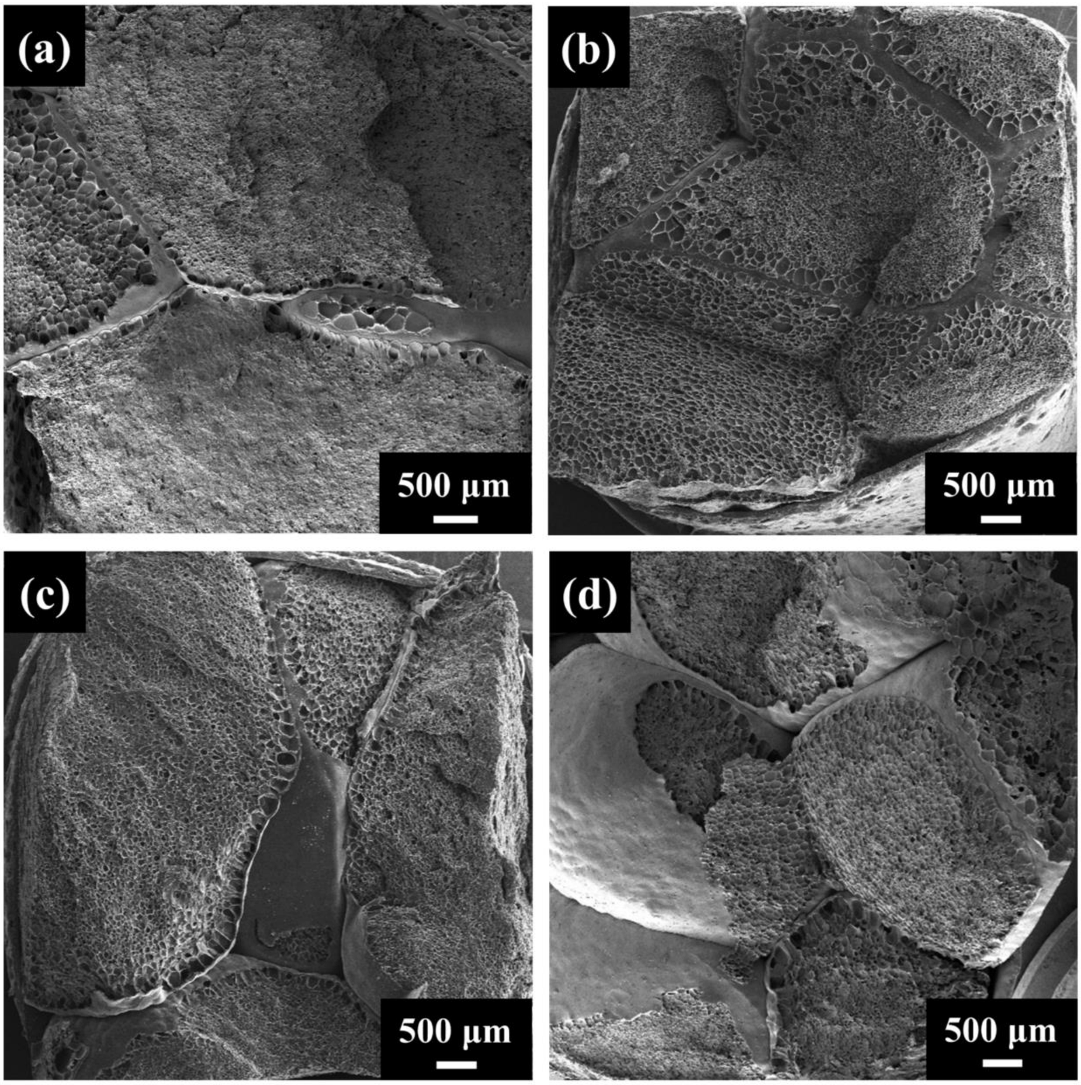


**Figure S1.** SEM image of the fracture surface of the ETPU tensile sample subjected to weathering aging process. (a) unaged sample; (b) aging 276 days; (c) aging 377 days; (d) aging 671 days.

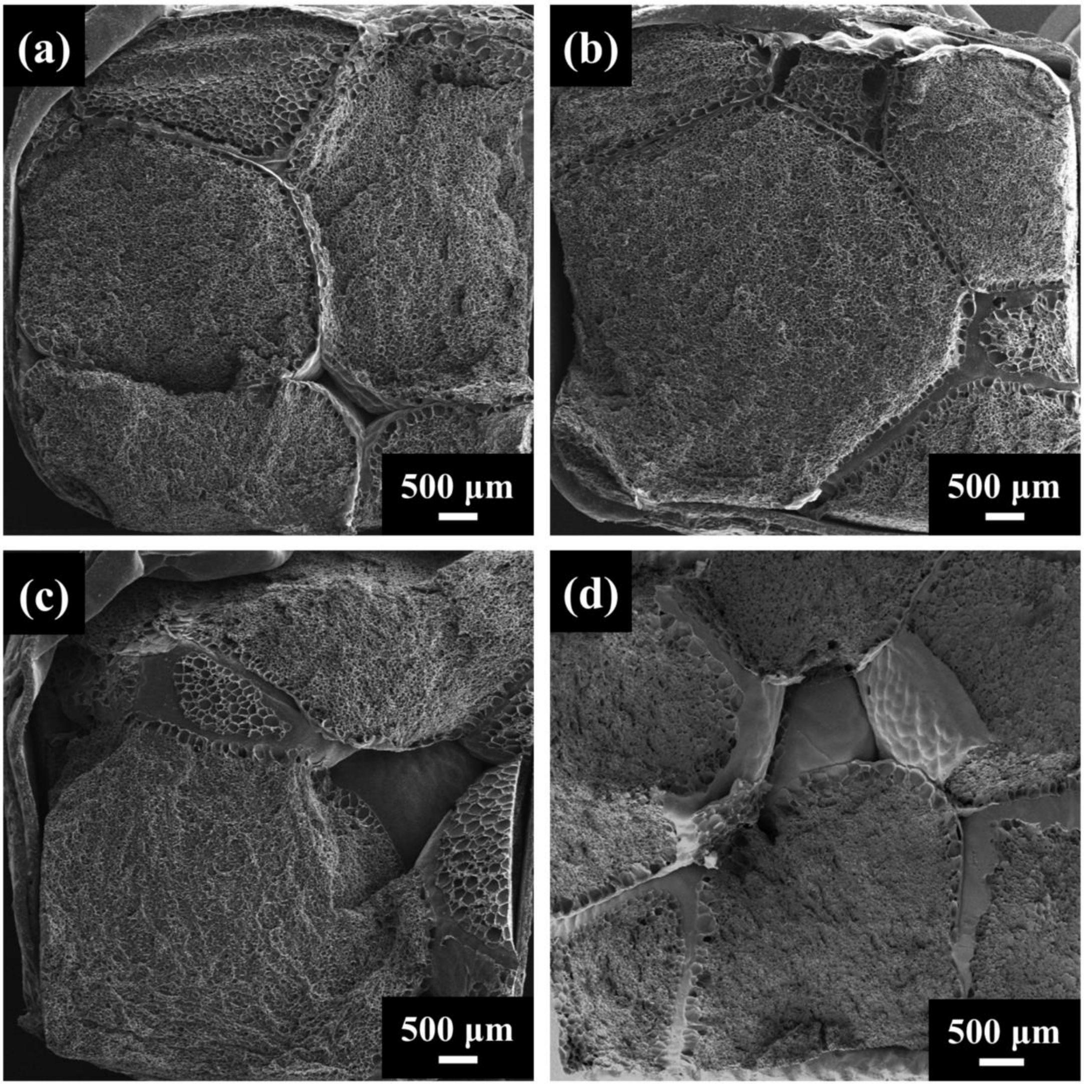


**Figure S2.** SEM image of the fracture surface of the ETPU tensile sample subjected to artificial thermal aging process. (a) aging 7 days; (b) aging 14 days; (c) aging 21 days; (d) aging 28 days.

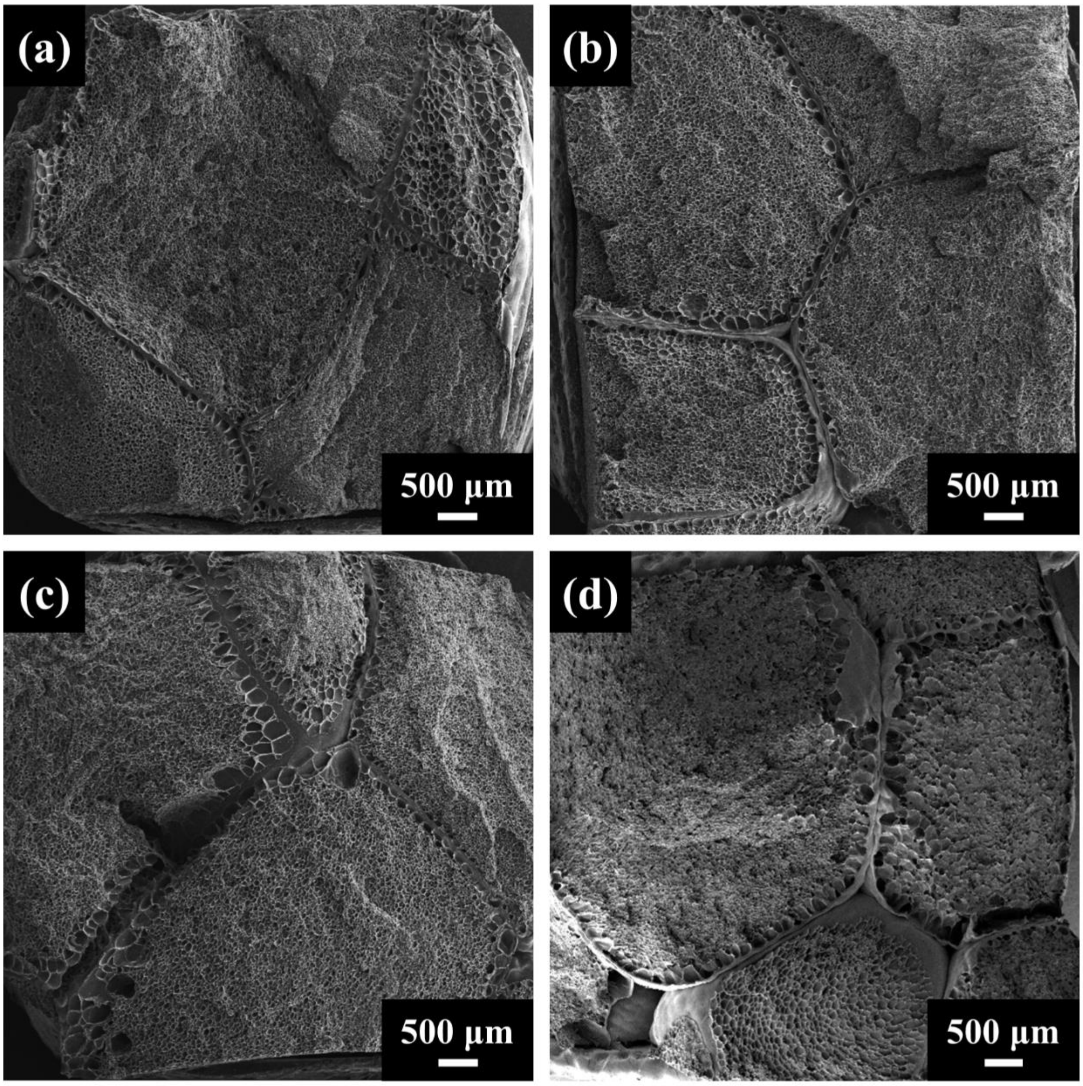


**Figure S3.** SEM image of the fracture surface of the ETPU tensile sample subjected to artificial xenon lamp aging process. (a) aging 7 days; (b) aging 14 days; (c) aging 21 days; (d) aging 28 days.
